# Supplementary figures and images for: Mapping Quantitative Trait Loci for 1000-Grain Weight in a Double Haploid Population of Common Wheat
Source: Int J Mol Sci. 2020 May 31;21(11):3960. doi: 10.3390/ijms21113960 (PMC7311974; doi:10.3390/ijms21113960)

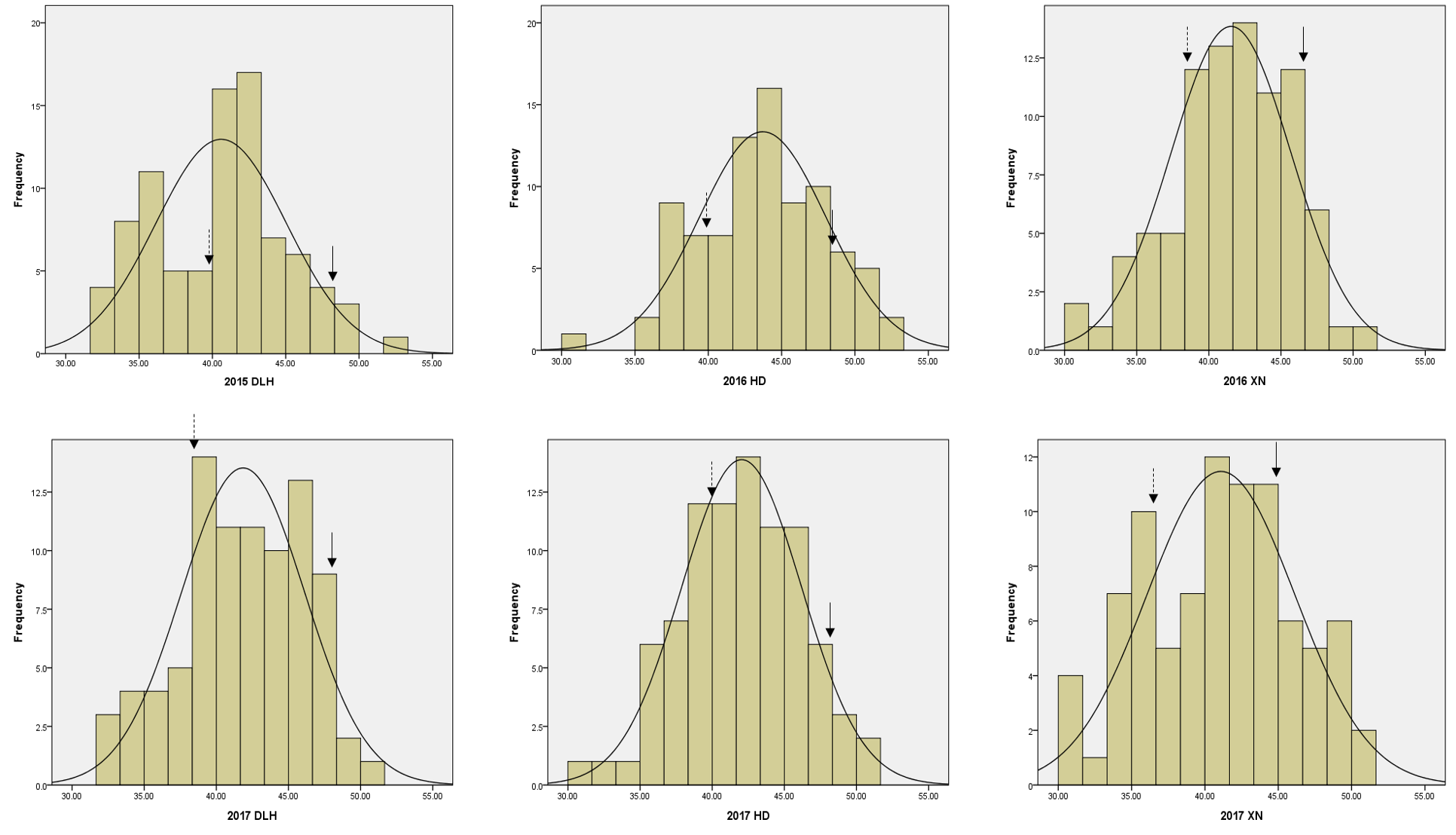

Supplement: Supplementary file 1 [file ijms-21-03960-s001.zip › Supplemental Fig. 2.tif]
